# A Large Cohort Study of Genotype and Phenotype Correlations of Beta- Thalassemia in Iranian Population

**Published:** 2015-10-01

**Authors:** Fereshteh Maryami, Azita Azarkeivan, Mohammad Sadegh Fallah, Sirous Zeinali

**Affiliations:** 1 Biotechnology Research Center, Department of Molecular Medicine, Pasteur Institute of Iran, Tehran, Iran; 2 Pediatric Hematology Oncology, Transfusion Research center, High Institute for Research and Education in Transfusion Medicine, Department of Thalassemia Clinic, Tehran, Iran; 3 Kawsar Human Genetics Research Center, Tehran, Iran

**Keywords:** α-thalassemia, β-thalassemia major, β-thalassemia intermedia, Xmn1 polymorphism, Iran

## Abstract

**Background:** Thalassemia syndromes are the most prevalent single gene disorders in Iran. This study aimed to evaluate the effect of different types of beta-globin gene mutations, co-inheritance of alpha-globin gene mutations and/or Xmn1 SNP on disease phenotype in a large cohort of Iranian patients.

**Subjects and Methods:** In total, 433 patients were clinically classified into β-thalassemia major (TM) or intermedia (TI). Multiplex PCR, ARMS-PCR, RFLP-PCR and DNA sequencing were performed to identify both α- and β-globin gene mutations and Xmn1 polymorphism as well. All data were compared and analyzed by SPSS software in TM and TI groups, consequently.

**Results:** A total of 39 different β-globin mutations were identified. Among them, the most common were IVS IInt1 (40.33%) followed by IVS Int5 (9.56%), C30 (7.22%) and Fr8-9(7%). All patients were subjected to evaluate common α-globin gene deletions. The patients inherited concomitant mutations of α- and β-globin, showed no clinical modifications compared with those who had only β-globin mutation. The TI patients showed a significant increase in frequency of both heterozygous and homozygous form of the Xmn1 polymorphism. It was also found that β^0^/β^0^ genotype patients, inherited the Xmn1 polymorphism required lesser blood transfusion.

**Conclusion:** No significant differences were observed, on the severity of disease, between patient's inherited defective α- and β-globin genes and ones with just β-globin gene mutation. Taking the results of this research into account, Xmn1 polymorphism can be considered as an important genetic factor modulating the severity of disease.

## Introduction

 Although, thalassemia is the most common monogenic disorders in Iran, but it is a very heterogeneous disease at the molecular and clinical levels.^1^ These variations depend on the extent of imbalances created between α- and non-α-globin chains synthesis.^2^^-^^3^ The incidence of β-thalassemia (β-thal) in Iran has been significantly decreased since 1997 due to the implementation of the National Program for the Prevention of Thalassemia.^4^^-^^6^ An average fall of about 81% has been reported for 2007-2009.^7^ The last available data about living patients, dated on 2007, revealed 13,879 thalassemia patients, with the mean age of 15 years old, all over the country.^6^ The carrier frequencies of β- and α-thalassemia (α-thal) are estimated to be 4-8%^8^ and 30%,^9^ respectively. Diagnosis of β-thal trait is suggested based on complete blood count (CBC) indices and hemoglobin (Hb) A2 level, although multiple factors like iron deficiency or α-thal trait should be taken into account.^10^ Moreover, It is claimed that co-inheritance of α- with β-hemoglobinopathies remarkably influences the clinical and hematological features of the disease. The later, might be related to the amount of α-globin chain deficiency which is associated with the type of the gene mutation.^11^

A wide range of α- and β-thal mutations has been detected among Iranian population. The most common α-globin gene mutation is -α^3.7^ kb^1^^,^^12^ followed by -α^4.2 ^kb, -α^20.5 ^kb and --Med deletions.^13^ Alpha-globin gene deletions account for more than 60% of α-globin mutations.^14^^-^^16^

The IVS IInt1 is reported to be the most common beta-globin gene mutation in Iran, followed by other point mutations depending on the population ethnicity.^6^^,^^17^^-^^18^ The frequency of α- and β- globin gene mutations vary throughout Iran notably from north to south.^19^^-^^21^

In previous studies, Xmn1-158 ^G^γ (C→T) (Xmn1 polymorphism) variant has been found to have an increasing effect on the HbF level, ameliorating the severity of the disease.^22^^-^^23^

This study aimed to evaluate factors affecting genotype and phenotype correlations in thalassemia major or intermedia such as co-inheritance of α-globin gene mutations and/or Xmn1 polymorphism in a large cohort study of Iranian β-thalassemic population. 

## SUBJECTS AND METHODS

**Subjects**

 A total of 433 patients suffering from either β-thal major or intermedia were admitted into this study. They were 200 males and 233 females; 32 children (≤12Y) and 401 adults (>12Y). Patients were referred to Kawsar Human Genetics Research Center by qualified hematologists from Thalassemia Clinics (Ethical committee No: 86022/14). According to clinical status, patients were classified into two groups: thalassemia major (TM) (n=89) and thalassemia intermedia (TI) (n=255).

The remaining patients (n=89) did not have any documented diagnosis. After obtaining informed consent, about 10 ml blood samples were collected in EDTA. DNA extracted via the standard salting out method,^29^ were quantified by nano drop spectrophotometer.

DNA genotyping ARMS-PCR method was employed to detect common β-globin genes mutations.^3^

DNA sequencing was done by Kawsar Biotech Co, Tehran, Iran (KBC), using BigDye Terminator Kit (Thermo Fisher Scientific Inc. Foster City, CA, USA, TF) and the samples were run on ABI 3130XL Genetic Analyzer (TF).

Common α-globin gene deletions (-α^3.7^, -α^4.2^, -α^20.5^ and --MED) were detected using a multiplex gap PCR method.^25^ Alpha-triplication was detected either by using PCR.^26^ Xmn1 polymorphism was investigated using RFLP-PCR method.^27^

**Statistical analysis**

All statistical analysis was performed with SPSS (version 16) (SPSS Inc., Chicago, IL, USA). Nonparametric Mann-whitney U test was used to compare TM and TI groups. Associations of β-thal with α-thal deletions and/or Xmn1 polymorphism were analyzed by nonparametric Chi-square and Kruskal-wallis (KW) test.

## Results

 A variety of factors were considered in our study design. Association of each factor with two groups of patients was taken into consideration. Table 1 provides a brief summary of measurements.

DNA samples were analyzed for α- and β-globin gene mutations by various molecular methods. A total of 39 different β-globin mutations were determined. IVS IInt1 was the most common detected mutation (40%) followed by IVS Int5 (10%), C30 (7%), and Fr8-9 (7%). The allele frequencies for different mutations are shown in Table 2.

Considering β-globin genotypes (i.e. β^++^, β^+^ and β^0^), patients were classified into different groups in accordance with criteria provided by Weatherall and Clegg.^3^ The data is presented in Table 3.

## Discussion

 According to the genotyping studies, the IVS IInt1 (G>A) was found as the most common mutation. All identified β-globin gene mutations (Table 2) are in a good agreement with most of the previous studies. 

**Table 1:** Clinical and molecular information of the β-thal patients

	**No** **(%)**	**Transfusion** ** dependency** (%**)**	**Mean** **age** **at first** **BT** **(months)**	**Transfusion ** **interval** ** (days)**	**Splenectomy ** **status** (%**)**	**Hydroxyurea ** **treatment** (%**)**	**BT ** **interruption ** **after ** **treatment** (%**)**	**Xmn1 polymorphism**			**Alpha genotype**		
								**+/+** (%**)**	**+/−** (%**)**	**−/−** (%**)**	**Mild** **α-thal** (%)	**Severe ** **α-** **thal** (%**)**	**H ** **disease** (%)
**TM**	89 (25.7)	85 (95.5)	24 ± 21	27 ± 24	39 (89.6)	4 (4.5)	0	12 (13.4)	37 (41.5)	39 (43.8)	8 (8.9)	4 (4.4)	0
**TI**	255 (73.9)	197 (77.2)	50 ± 38	30 ± 19	128 (50.1)	60 (23.5)	23 (38.3)	101 (39.6)	87 (34.1)	67 (26.2)	18 (7.0)	2 (0.7)	1 (0.3)

**TM:** Thalassemia Major, **TI:** Thalassemia Intermedia, **BT:** Blood Transfusion, **Xmn1:** -158 Gγ (C→T) (Xmn1 is a restriction polymorphic site with known association with the severity of the disease)

**Table 2:** β-globin mutations in the β-thal patients

**Mutation**	**HGVS** **nomenclature**	**Total ** **allele ** **(n)**	**Allele ** **frequency ** **(%)**
**IVS II-1 G→A**	HBB: c.315+1 G>A	353	40.33 %
**IVS I-5 G→C**	HBB: c.92+5 G>C	83	9.56 %
**CD30 G→C**	HBB: c.93 G>C	63	7.22 %
**CD8-9 + G**	HBB: c.27_28 ins G	61	7.00 %
**IVS I-110 G→A**	HBB: c.93-21 G>A	43	4.89 %
**CD 36-37 −T**	HBB: c.112 del T	33	3.78 %
**IVS I-6 T→C**	HBB: c.92+6 T>C	23	2.67 %
**CD5 − CT**	HBB: c.17_18 del CT	26	3.00 %
**CD44 − C**	HBB: c.135 delC	21	2.44 %
**IVS I-1 G → T**	HBB: c.92+1G>T	17	1.89 %
**-88 C → T**	HBB: c.-138 C>T	12	1.33 %
**IVS I (-25 bp del)**	HBB: c.93-21-96 del	10	1.11 %
**CD 22 (-7 bp del)**	**----**	22	2.56 %
**Others** **(26 mutations)**	------	95	10.89 %
**unknown**	----------	4	0.46%
**Total chromosome**		866	100%

**HGVS: **Human Genome Variation Society, **IVS: **Intervening Sequence (Intron) (mutations in the introns of beta-globin are shown by IVS), **HBB: **Beta-globin gene,** CD: **Codon

Geographic distribution of population in Iran could be considered as the main reason of discordances between our study and other related researches.^6^^, ^^17^^-^^18^ No mutation was detected in 0.5% of β-thal alleles (n=4) neither by ARMS-PCR nor direct sequencing. However, two samples of them revealed anti 3.7 ααα through performing related PCR. Therefore, just a value of 0.25% of mutations could not be determined via the former methods. Association of Xmn1 and blood transfusion frequency has already been reported by Winichagoon et al.^28^ Our research showed higher frequency of Xmn1 polymorphism (+/+ or -/+) in TI compared to TM patients (p<0.0001). Moreover, it is observed that non transfusion dependent thalassemia (NTDT) patients with β^0^/β^0^ genotype, inherited Xmn1 polymorphism (p<0.0001). Sivalingam et al.^29^ have reported that patients carrying Xmn1 polymorphism required less frequent transfusion.

Dedousis et al.^30^ have studied on the presence of clinical variability in patients who were homozygote or compound heterozygote for β^0^ or β^+^ thalassemia. They have found that rising fetal hemoglobin (HbF) level improved the clinical feature of the disease. Increasing of HbF has been attributed to the association of some β-globin mutations with the Xmn1 polymorphism. Pandey et al.^   23 ^ have reported 

**Table 3:** Frequencies of different genotypes

	**β** ^N^ **/β** ^N^	**β** ^0^ **/β** ^N^	**β** ^0^ **/β** ^unknown^	**β** ^0^ **/β** ^0^	**β** ^0^ **/β** ^+^	**β** ^0^ **/β** ^++^	**β** ^+^ **/β** ^+^	**Hbvar/Hbvar**
**TM**	0	0	1 (1.1%)	53 (59.5%)	11 (12.3%)	2 (2.2%)	12 (13.4%)	1 (1.1%) (δβ/δβ)
**TI**	1 (0.3%) (H-dis)	2 (0.7%) (anti-3.7)	1 (0.3%)	180 (71.3%)	39 (15.2%)	2 (0.7%)	26 (10.1%)	2 (0.7%) (HbS/HbS)

**TM:** Thalassemia major, **TI:** Thalassemia Intermedia,** HbVar:** Hemoglobin variant

that HbS-β thalassemia patients were clinically variable, ranging from a completely asymptomatic to a severe disorder. Others suggested that this heterogeneity could be caused by either different β-thal mutations or interactions between different genetic modulating factors like co-existence of α-thal and/or Xmn1 polymorphism.^   31 ^

It has been reported that co-inheritance of α-thal could improve the clinical severity in β-thal patients.^32^ It is not possible to confirm that through this study. This discordance could be due to the lower frequency (10%) of α-globin gene deletions detected in the individuals. Different α-globin gene mutations have been shown to be prevalent in Iran accounting up to 30%,^9^ among them, α-globin gene deletions account for more than 60% of α-globin mutations.^14^^-^^16^ It was surprising to find lesser frequencies of α-deletions in our individuals.

Based on the results of this research, genotype determination is beneficial for early prognosis of β-thal and to choose the best possible treatment. Moreover, Xmn1 polymorphism has shown more ameliorating effect on the phenotype of our patients.
